# Integrated Welfare Monitoring in Laying Hens: A Systematic Literature Review, Expert Insights and a Camera Proof-of-Concept

**DOI:** 10.3390/ani16172794

**Published:** 2026-09-05

**Authors:** Sam Willems, Amélie Canon, Hanne Coppens, Niels Demaître, Nathalie Sleeckx, Tomas Norton

**Affiliations:** 1 M3-BIORES Group, Division Animal and Human Health Engineering, Department of Biosystems, Catholic University of Leuven, Kasteelpark Arenberg 30, 3001 Heverlee, Belgium; sam.willems@kuleuven.be (S.W.); amelie.canon@kuleuven.be (A.C.); hanne.coppens@vencomaticgroup.com (H.C.); 2 Vencomatic Group, Meerheide 200, 5521 DW Eersel, The Netherlands; 3 Experimental Poultry Centre of Province of Antwerp, Poiel 77, 2440 Geel, Belgium; niels.demaitre@provincieantwerpen.be (N.D.); nathalie.sleeckx@provincieantwerpen.be (N.S.)

**Keywords:** laying hens, automated welfare monitoring, pan-tilt-zoom cameras, precision livestock farming, feather pecking, expert opinions, commercialisation

## Abstract

Effective welfare monitoring in laying hens is increasingly challenged by growing flock sizes, declining farm numbers, and the practical limitations of assessor-based protocols under commercial conditions. Precision Livestock Farming (PLF) technologies offer opportunities to support large-scale welfare assessment, yet their application in laying hens remains predominantly limited to small-scale or prototype systems. This study addresses three complementary objectives aimed at informing future computer-vision-based PLF research and development in commercially housed laying hens. First, a systematic literature review was conducted to identify welfare-related categories monitored in laying hens between 2005 and 2025, the methods used to assess them, and the extent to which automated monitoring approaches have been applied. Second, outcomes of a TransRegional Expert Panel (TREP) within the OMELETTE project were synthesised to rank priority welfare challenges, evaluate the feasibility of different monitoring approaches, and compare expert perspectives with trends identified in the literature. Third, two pan-tilt-zoom (PTZ) camera setups implemented in semi-commercial aviary systems were described as proof-of-concept examples illustrating how multiple welfare challenges can be monitored using a single, multipurpose camera system. The literature review revealed a pronounced imbalance in monitoring frequency, with feather pecking dominating the literature while several other welfare challenges, including piling, disturbed sleep, and toe-pecking, remain comparatively underrepresented. TREP outcomes confirmed feather pecking as the highest-priority welfare challenge but also highlighted the importance of integrated monitoring approaches that combine time-intensive assessments, shorter checklists, and automated systems rather than relying on single indicators. Experts further considered the use of digital devices during routine barn inspections to be practically feasible. The PTZ proof-of-concept demonstrates how priority welfare challenges identified through both literature and expert input can be operationalised through automated, scheduled, and location-specific monitoring under commercial conditions. Together, these findings highlight the need for future PLF research to move beyond isolated measurements and small-scale trials towards integrated, cost-effective, and farm-specific welfare-monitoring systems that support adaptive, data-informed management strategies—for example, within a Plan–Do–Check–Act framework—and enable the development of digital standard operating procedures that generate actionable insights under real-world commercial constraints.

## Simple Summary

Monitoring the welfare of laying hens is becoming more difficult due to increasing flock sizes and the practical limits of manual inspections on commercial farms. New technologies, especially camera-based precision livestock farming tools, could help farmers monitor animal welfare more efficiently, but their use is still limited in practice. This study combined three approaches to better understand how welfare monitoring can be improved. First, a review of the scientific literature showed that most research focuses heavily on feather pecking, while other important welfare problems, such as piling and sleep disturbances, are much less studied. Second, experts from across the egg-laying sector confirmed that feather pecking is a key concern, but also emphasised the need to monitor multiple welfare issues together and to combine manual checks with automated tools. Third, we described a camera-based system that can monitor several welfare indicators using a single device, demonstrating how such technologies could be applied on farms. Overall, the findings highlight the need for more integrated, practical, and cost-effective monitoring approaches that combine different data sources and support farmers in making better decisions to improve animal welfare under real farm conditions.

## 1. Introduction

Flock sizes are increasing, farm numbers are declining, and the reliance on manual welfare monitoring has become increasingly impractical at commercial scale [1]. Under these conditions, automated monitoring using Precision Livestock Farming (PLF) technologies has been proposed as a necessary alternative to support large-scale welfare assessment. In 2024, laying hens accounted for approximately 8.65 billion birds globally and produced nearly 1.8 trillion eggs annually, highlighting the continued expansion of the sector [2]. Despite this growth, PLF in poultry remains underdeveloped compared to sectors such as dairy farming, where commercially successful systems such as automated milking and oestrus detection have been established for decades [3]. Indeed, 96.21% of identified poultry PLF systems remain at the prototype stage, with only a limited number of commercial solutions available [3].

Commercialisation of PLF technologies has been hindered by several persistent barriers, including limited early involvement of manufacturing companies [4], a lack of large-scale on-farm commercial trials [4], and farmer uncertainty regarding return on investment and confidence in the technology [5]. More recent studies confirm that although motivation to adopt PLF exists, it is often tempered by unproven benefits, pricing concerns, doubts regarding maintenance services, and uncertainties related to data ownership, access, and rural internet connectivity [6,7]. Taken together, these barriers suggest that the challenge extends beyond technical development to the ability of PLF systems to generate sustained and demonstrable value under commercial farm conditions.

Addressing this gap requires research that moves beyond small-scale experimental settings towards welfare-monitoring tools designed, evaluated, and refined under real farm conditions, as results obtained in controlled environments do not readily translate to commercial practice. Effective welfare management in laying hen systems relies heavily on established assessor-based protocols, including Welfare Quality [8] and AssureWel [9]. Laying hen welfare is inherently multifactorial, and studies commonly assess a primary welfare outcome alongside complementary animal-based, production-based, management-based, or resource-based measures to provide a broader interpretation of welfare. While these frameworks are structured and validated, their reliance on trained assessors and time-intensive procedures limits routine application at commercial scale. More time-efficient alternatives, such as the Aviary Transect method [10], and digital tools including the “Dierenwelzijns scan” [11] and the EBENE app [12], facilitate data recording and benchmarking, yet remain inherently intermittent and assessor-dependent. Consequently, welfare is typically captured at discrete moments rather than continuously, and it often remains unclear how recorded data translate into actionable management decisions that improve hen health, longevity, and productivity. These limitations align with earlier observations by [5] and the work of [13,14], who highlighted persistent uncertainty regarding return on investment and a lack of practical on-farm evidence demonstrating how PLF outputs can be embedded into routine management workflows.

Several commercial data platforms aim to support farmers by structuring and visualising specific data streams. Poultry Plan [15], for example, integrates production and management data and enables benchmarking against breed-specific reference curves, while Meggsius Connect [16] focuses on automated collection and visualisation of egg production and egg quality data. Although these platforms provide valuable operational support, they typically focus on isolated subsets of data rather than offering an integrated overview of animal health and welfare. In complex and dynamic laying hen housing systems, production and welfare outcomes are shaped by multiple interacting factors, and single indicators rarely provide sufficient explanatory power in isolation. This is consistent with findings by [7,17], who demonstrated that management strategies and technology performance are highly farm-specific. In the context of poultry red mite (PRM) control, ref. [17] showed that Integrated Pest Management strategies must be adapted to farm-specific conditions, while [7] reported that relationships between environmental variables and production outcomes vary substantially between farms.

Accordingly, no universal solution can simultaneously guarantee hen welfare, productivity, longevity, and farmer profitability across farms. Welfare management therefore requires adaptive approaches that explicitly account for farm-specific dynamics. Iterative management frameworks such as the Plan–Do–Check–Act (PDCA) cycle [18] provide a useful conceptual basis, treating management decisions as testable hypotheses rather than fixed prescriptions. Within such frameworks, continuous or high-frequency monitoring enables repeated evaluation and refinement of management actions over time, aligning well with the core principles of PLF.

Taken together, these considerations highlight the need for an integrated perspective that avoids fragmented interpretation of welfare-related information and supports timely, effective interventions in commercial laying hen systems. While existing platforms successfully integrate specific data streams, they do not yet provide a comprehensive framework that combines production data, manual welfare assessments, environmental information, and automated welfare measurements in a single coherent and interpretable manner. From a PLF perspective, integrating multiple automated welfare measurements within one system is therefore a crucial step towards meaningful, continuous, and farm-specific welfare monitoring.

Against this background, this paper pursues three main objectives. First, a systematic literature review is conducted to identify welfare-related categories and monitoring approaches in laying hens, addressing the following research questions:

(1) Which welfare-related categories in laying hens have been monitored between 2005 and 2025, and how frequently has each category been investigated?
(2) Which monitoring methods have been used to assess these welfare-related categories?
(3) To what extent have automated monitoring tools been applied, and where do opportunities lie for PLF technologies?

Second, to complement the scientific literature with sector perspectives relevant to commercial implementation, outcomes of the TransRegional Expert Panel (TREP) of the OMELETTE project [19] are synthesised. Experts from across the European egg sector ranked priority welfare challenges and evaluated the feasibility of different monitoring approaches, and these perspectives are compared with trends identified in the literature.

Third, informed by both the literature review and expert input, two pan-tilt-zoom (PTZ) camera monitoring setups implemented within the OMELETTE project are described as proof-of-concept examples for computer-vision-based PLF research in commercially housed laying hens. Both setups make use of PTZ cameras to monitor multiple welfare-related challenges with the same camera device. To our knowledge, the use of PTZ cameras for this type of integrated welfare monitoring has not previously been investigated in commercially housed laying hens, representing a novel application of computer vision within this context.

Together, these three main objectives aim to inform future PLF research that moves beyond small-scale studies towards integrated, cost-effective, and farm-specific welfare-monitoring systems capable of supporting adaptive, data-informed, PDCA-like management strategies under commercial conditions.

This study provides a novel integration of a systematic literature review on laying hens, structured expert input, and a real-world proof-of-concept to support the development of integrated welfare-monitoring strategies for commercial housing systems.

## 2. Materials and Methods

### 2.1. Systematic Literature Review

#### 2.1.1. Literature Search

**Figure 1:**
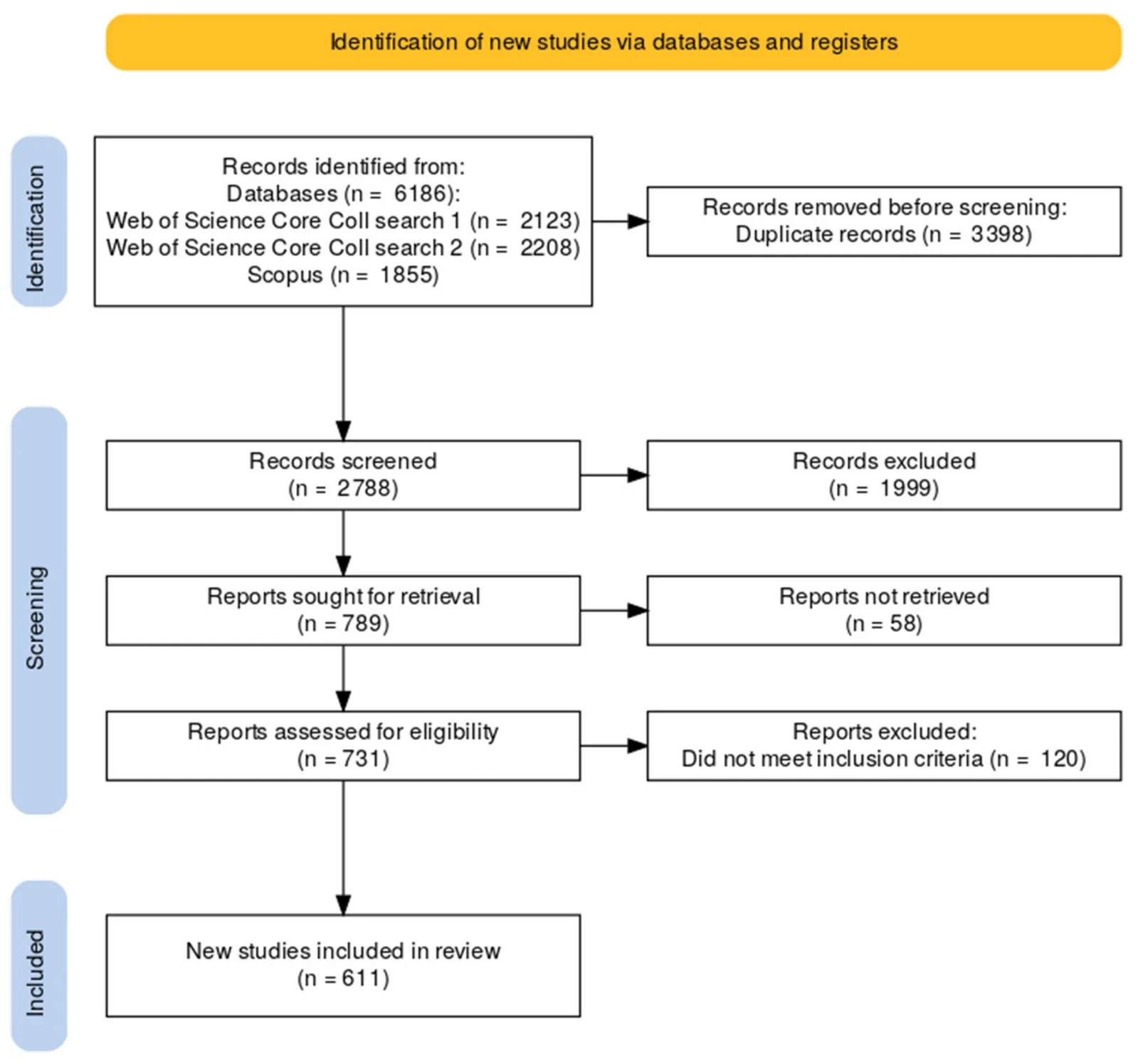
PRISMA flowchart.

A systematic literature search was conducted in line with PRISMA guidelines to identify original research publications (see Figure 1). The focus was on studies applying manual or automated monitoring approaches to investigate welfare challenges in laying hens, with particular emphasis on animal-based indicators, as defined within established welfare assessment frameworks [8]. Searches were carried out using the Web of Science Core Collection and Scopus databases. Two complementary search strategies were applied in both databases to capture both welfare challenges in laying hens and the technologies used to monitor them.

#### 2.1.2. Selection of Search Terms

The first search focused on welfare-related problems in laying hens. Search terms were derived from established assessment protocols, including Welfare Quality [8], AssureWel [9], and the Aviary Transect protocol [10]. Together, these protocols provided a comprehensive foundation for identifying a broad set of welfare issues, which were then complemented with laying-hen-specific terms. To ensure comprehensive coverage, synonyms and closely related expressions were also included to capture variations in terminology across studies. This first search was performed to retrieve articles in which welfare challenges were monitored either manually or automatically.

The second search focused only on automated monitoring approaches. Here, terms were adapted from [3] but narrowed to concentrate exclusively on laying hens. The final set included terms associated with PLF, sensor technologies, acoustic monitoring, thermal imaging, and computer vision, supplemented with synonyms and spelling variations where applicable.

In both searches, quotation marks were applied to multi-word expressions (e.g., “egg eating”) to restrict results to exact phrases and increase specificity. Wildcards such as the asterisk were used to capture variations in root words (e.g., peck* to include peck, pecks, pecking), and question marks were applied where a single character could vary between different spellings (e.g., “vocali?ation” to capture both vocalization and vocalisation).

#### 2.1.3. Search Strategy

Search 1 (welfare-related terms). For the first search, a topic search (TS) was conducted to retrieve English-language articles published between 2005 and 2025. Welfare-related search terms extracted from the assessment protocols described in Section 2.1.2 were combined using the OR operator and further combined with laying-hen-specific terms using the AND operator. In the Web of Science Core Collection, two searches were conducted. In the first search, only original research articles were included (DT = Article, where DT refers to document type), while review papers were excluded (NOT DT = Review). A second Web of Science Core Collection search was subsequently conducted without restrictions on publication type by omitting the line “DT = (Article) NOT DT = (Review)”, thereby retrieving all publication types, including conference papers. In Scopus, one search was conducted without restrictions on publication type.

The search string was constructed using search terms presented in Table 1.

Search 2 (automated monitoring terms). For the second search, a topic search (TS) was conducted. Automated monitoring search terms adapted from [3], as described in Section 2.1.2, were combined using the OR operator and further combined with laying-hen-specific terms using the AND operator. In the Web of Science Core Collection, two searches were conducted. The first search included only original research articles, while the second search placed no restrictions on publication type. In Scopus, one search was conducted without restrictions on publication type.

The search string was constructed using search terms presented in Table 2.

#### 2.1.4. Eligibility Screening

In this review, the term “production outcomes” refers to measures directly related to productivity and product quality, including feed intake, feed conversion ratio, egg production, egg mass, egg weight, eggshell strength, and yolk or albumen quality. In general, studies that focused exclusively on production outcomes without addressing behaviour or welfare were excluded. An exception was made for studies on PRM infestation and comparable welfare threats, where manual or automated monitoring linked to production outcomes was also considered eligible, as these threats are often studied primarily through their impact on hen performance. Specifically for PRM, prevalence rates above 80% have frequently been reported across European countries [20,21]. Given its widespread occurrence and relevance for both hen welfare and farm productivity, PRM was considered an important welfare challenge to include in this review, thereby justifying the exception made for this welfare challenge.

Studies were included if they:

- Were original journal articles or conference papers;
- Monitored at least one other welfare problem or closely related concept listed in Search 1 (i.e., conceptually equivalent or synonymous terms) in live laying hens;
- Used non-invasive methods (e.g., direct behavioural observation, scoring protocols, video or camera-based monitoring, floor egg counts, mortality records, environmental sensors);
- Used minimally intrusive methods (e.g., wearable sensors such as RFID tags, accelerometers, GPS devices, or palpation-based keel bone scoring);
- Exception for studies on PRM infestation and comparable welfare threats: Articles monitoring PRM or comparable threats, either manually or automatically, were included when this monitoring was explicitly linked to at least one other welfare problem or closely related concept listed in Search 1 (e.g., conceptually equivalent or synonymous terms) in laying hens, or to production outcomes (as described above).

Studies were excluded if they:

- Were not original journal articles or conference papers (e.g., review articles, meta-analyses, book chapters, meeting abstracts, letters);
- Focused exclusively on production outcomes without addressing behaviour or welfare, except for studies on PRM infestation and comparable welfare threats as described above;
- Investigated only post-mortem outcomes (e.g., carcass dissection, tissue sampling, bone strength tests) or included only deceased hens;
- Investigated other poultry categories (e.g., broilers, broiler breeders, pullets, layer breeders, roosters, or other avian species such as ducks, turkeys and quail);
- Relied primarily on physiological or laboratory-based measures (e.g., blood haematology, hormone assays, biomarkers, histology, gene expression) without addressing behaviour or welfare;
- Reported welfare indicators (e.g., mortality, floor eggs) only as incidental outcomes in nutrition, toxicology, or physiology studies (e.g., describing symptoms of diseases, such as inactivity) rather than as part of monitoring unwanted behaviours or welfare problems;
- Monitored PRM infestation or a comparable welfare threat only, without linking its monitoring to at least one other welfare problem or closely related concept listed in Search 1 (e.g., conceptually equivalent or synonymous terms) in laying hens, or to production outcomes.

All 2788 records remaining after duplicate removal were screened by Reviewer 1 (SW) using Rayyan Pro software [22]. Rayyan Pro supports automated duplicate removal when titles matched exactly, abstract screening, conflict resolution, full-text screening, and data extraction in a double-blind manner. Reviewer 2 (HC) independently screened a random subset of 16% of the records while remaining blinded to the screening decisions of Reviewer 1. A total of 44 conflicts were identified. Of these, 29 involved one reviewer selecting “include” while the other selected “exclude”, whereas 15 involved one reviewer selecting “indecisive” and the other selecting either “include” or “exclude”. All conflicts were resolved through discussion by reassessing the article against the predefined inclusion and exclusion criteria described above. In cases of uncertainty, reviewers jointly evaluated whether the study population, study design, monitored outcome, and relevance to laying hen welfare met the eligibility criteria. Articles marked as indecisive by one reviewer were ultimately agreed upon by both reviewers to be excluded. Following title and abstract screening, 789 reports were sought for full-text retrieval. Of these, 58 could not be retrieved, leaving 731 reports for full-text eligibility assessment. In total, 120 reports were excluded during the full-text assessment and data extraction process because they did not meet the predefined eligibility criteria, resulting in 611 articles being included in the final review.

Eligibility assessment was performed by reading each full-text article in detail. Inclusion and exclusion decisions were made simultaneously with the data extraction process.

#### 2.1.5. Data Extraction

For each welfare indicator or welfare challenge reported in an article, three pieces of information were extracted: (i) the original terminology used by the authors to describe the welfare challenge, (ii) the monitoring method describing how the welfare challenge was investigated or monitored in this study, and (iii) the predefined welfare-related category that most closely matched the welfare challenge addressed (see description below for the predefined welfare-related categories). Monitoring methods were subsequently classified as (i) manual bird-based assessment, (ii) manual assessment not based on birds, (iii) automated monitoring using algorithms or dedicated devices, or (iv) visual observation from images or videos.

During the initial phase of the review, Reviewer 1 (SW) extracted data from the first 100 articles and recorded the original terminology used by the authors to describe welfare challenges and welfare indicators. Based on these first 100 articles, a preliminary classification framework consisting of 27 welfare-related categories was developed. This initial framework was subsequently used throughout the remainder of the data extraction process. Using the 27 welfare-related categories, Reviewer 3 (AC) assessed and extracted data from 444 articles. Articles excluded by Reviewer 3 (AC) were independently reviewed by Reviewer 2 (HC). Any remaining conflicts were subsequently checked by Reviewer 1 (SW) and discussed among the reviewers until consensus was reached. Reviewer 1 (SW) assessed and extracted data from the last 187 articles. Following the full-text assessment and data extraction process, 611 unique articles met all eligibility criteria and were included in the final review. Following completion of the extraction process, Reviewer 1 (SW) systematically reviewed all extracted data from all included articles. To facilitate this process, lists containing the original terminology used by the authors were generated for each predefined welfare-related category. These lists were closely examined to identify inconsistencies and potential misclassifications, and were corrected in this way. Subsequently, the initial framework consisting of 27 welfare-related categories was refined into a final framework consisting of 25 welfare-related categories, which were grouped into 7 super-categories. Table 3 presents the 7 super-categories and their corresponding 25 welfare-related categories, together with examples of the original terminology used by the authors, to classify welfare challenges into each welfare-related category. To assess consistency in data extraction between reviewers, Reviewer 3 (AC) independently performed blinded data extraction for 49 randomly selected articles that had also been extracted by Reviewer 1 (SW). Across these articles, Reviewer 1 identified 165 welfare challenges, whereas Reviewer 3 identified 172. Agreement between reviewers was high, with a Cohen’s kappa of 0.939 for the extracted welfare challenges and 0.934 for the corresponding monitoring methods. A detailed overview of the double-blind data extraction for these 49 articles is available in our Open Science Framework repository (https://osf.io/4hkx9/, accessed on 1 September 2026).

### 2.2. TransRegional Expert Panel (TREP) Meeting

The OMELETTE project operates in North-West Europe (NWE), a major egg-producing region with wide variation in housing systems, welfare standards, and adoption of digital tools. We established a TREP that brought together stakeholders across the laying hen industry, including farmers and sector organisations, veterinarians, SMEs/industry, public authorities, research and higher education, NGOs, retailers, and consumer organisations. Participants were not recruited through a formal stratified sampling procedure. Instead, invitations were distributed across a broad range of stakeholder groups relevant to the laying hen sector, and participation was based on attendance at the TREP workshop rather than on predefined sampling strata or target proportions. In total, 42 associated organisations from NWE were represented by 45 participants who joined an interactive workshop and provided informed consent for the anonymous use of their votes and discussion contributions. Country information was available for 34 participants. Of these, 15 were from Belgium, six from the Netherlands, five from France, three from Germany, two from Ireland, and one each from Switzerland and the United Kingdom; one participant reported another country.

#### 2.2.1. Format and Facilitation

A TREP meeting was organised at the start of the project. Participants were evenly distributed across three parallel breakout rooms, each led by a moderator and supported by a notetaker. We ensured that each room contained a balanced mix of stakeholder types and avoided placing multiple participants from the same organisation in the same room. An interactive PowerPoint with embedded Slido^®^ software version 1.9.1.5104 (Slido, Bratislava, Slovakia) was used to collect real-time votes and immediately display aggregated results, which then guided short, structured discussions. Notetakers documented key discussion points throughout.

#### 2.2.2. Interactive TREP Meeting

Before the meeting, OMELETTE project partners independently compiled a non-exhaustive list of 24 welfare problems in laying hens, including egg eating, cannibalism, feather pecking, fearfulness and flightiness, piling, excessive aggression, stereotypies and boredom, lethargy, toe-pecking, keel bone damage or deformation, mortality, floor eggs, heat stress, disturbed sleep, respiratory and eye problems, aberrant vocalisations, foot, body, or head injuries, antagonistic behaviours, feather loss, beak abnormalities, and footpad lesions. This list was developed separately from the systematic literature review and was not derived from its search terms or welfare-related categories. Using Slido, partners ranked these welfare problems according to their perceived impact on hen longevity. Based on the outcomes of this ranking, seven unwanted behaviours were subsequently pre-selected for in-depth discussion within the TREP: feather pecking, fearfulness and flightiness, atypical egg laying, stereotypic behaviours and boredom, piling, toe-pecking, and disturbed sleeping patterns. These behaviours were selected because they were considered particularly relevant from a practical perspective and in relation to current behavioural and welfare concerns in laying hens. Limiting the selection to seven behaviours also provided a clear and manageable scope for the workshop, allowing for more in-depth discussion rather than covering the full range of possible behavioural indicators.

These seven behaviours formed the basis of the questions presented in the breakout rooms via the Slido-enabled PowerPoint presentation. Printed cards listing the seven behaviours were provided to all participants to facilitate quick reference during voting and discussion. For the present study, the quantitative TREP analysis focused on two topics:

(Topic 1) Unwanted behaviours in laying hens: Five ranking questions were included in the analysis.

(Topic 2) Monitoring approaches (manual scoring and automated/PLF tools): Five multiple-choice questions were included in the analysis.

An overview of the questions included in the present analysis is provided in Table 4.

#### 2.2.3. Compilation of Responses and Data Availability

Poll results were compiled at the breakout-room level to provide an overview of priorities and perspectives regarding welfare problems and monitoring strategies. The room-level outputs, notetakers’ summaries, and a complete Slido-enabled PowerPoint presentation are available in our Open Science Framework repository (https://osf.io/4hkx9/, accessed on 1 September 2026).

### 2.3. Proof-of-Concept: PTZ Camera Setup for Automated Monitoring in OMELETTE

To complement the systematic literature review and expert panel discussions, a camera-based monitoring setup was developed as a proof-of-concept for future computer-vision-based PLF research in commercially housed laying hens. This setup illustrates how a limited number of pan-tilt-zoom (PTZ) cameras can be configured to monitor multiple priority welfare challenges using preconfigured camera presets and automated scheduling. PTZ cameras enable automated adjustment of the field of view throughout the day and night, allowing a single camera device to monitor different locations within the laying hen house. Within the OMELETTE project, cameras were configured to support daily monitoring of individual feather condition, piling behaviour, floor egg or out-of-nest egg occurrence, and nighttime activity patterns by following a predefined daily recording schedule and PTZ preset sequence. During the lights-on period, cameras captured still images every 90 s, whereas during the lights-off period, they recorded videos at a resolution of 1920 × 1080 pixels at 15 frames per second.

As no quantitative evidence was generated in this study, the setup is described solely as an illustrative example of how integrated, automated welfare monitoring could be implemented under semi-commercial conditions at the Experimental Poultry Centre (EPC). Two test cases were considered. The first test case involved a single compartment consisting of two experimental units, each housing 1325 Warren Brown laying hens in a two-row multi-tier aviary system without outdoor access. Only one of the experimental units was equipped with two PTZ cameras and included in the monitoring study. The second test case involved a separate compartment, also consisting of two experimental units, each housing 960 Dekalb White laying hens in a single-row multi-tier aviary system without outdoor access. In this case, each experimental unit was equipped with six PTZ cameras. As the monitored experimental units had comparable dimensions, the primary difference between the two test cases was the number of deployed cameras. Consequently, the first test case required more extensive use of zoom levels and camera presets to obtain sufficiently detailed views of the different monitoring locations.

The recording schedule was aligned with farm management practices and the expected visibility and relevance of the targeted welfare indicators. Between 04:00 and 07:00, cameras were configured for floor egg monitoring before routine egg collection by farm staff. Piling monitoring was scheduled between 07:00 and 09:00, 11:00 and 14:00, and 15:00 and 17:00 to obtain coverage across different periods of the day. Feather scoring was scheduled between 09:00 and 11:00 and between 14:00 and 15:00, corresponding to feeding periods when a larger proportion of hens were positioned on the aviary system and therefore visible for individual assessment. An additional feather-scoring period was included between 17:00 and 18:00, when hens increasingly moved towards the aviary system in preparation for the lights-off period. Nighttime videos were recorded between 21:00 and 00:00 for sleep and PRM-related monitoring. This period was selected within the middle part of the dark phase, consistent with previous work highlighting this period as particularly informative for monitoring nighttime activity and sleep-related disturbances [23].

The recording infrastructure was designed to be cost-effective and suitable for daily on-farm recording and comprised low-cost, commercially available Power-over-Ethernet (PoE) cameras, CAT6 Ethernet cabling, a managed network switch, a network-attached storage device for local data handling, and a standard home internet connection (approximately 25 Mbps) for data transfer to external servers. Every night at 01:30, approximately 90 gigabytes of data are uploaded to external servers, taking around eight hours to complete, enabling automated daily analysis of the recorded data.

Within the OMELETTE project, an automated pipeline to process these daily recordings is being implemented in GitLab using a CI/CD pipeline and a GitLab runner installed on a dedicated Linux machine equipped with an NVIDIA GPU. Neural networks were trained to address the different welfare challenges, with separate training, validation, and test datasets constructed for each task. YOLOv8 object detection models were trained to detect (1) individual hens in zoomed-in images of the aviary system for feather scoring in both test cases, (2) individual hens in zoomed-out nighttime images for nighttime monitoring, (3) floor eggs in zoomed-out images of the litter area in the second test case and in zoomed-in images of the litter area in the first test case, and (4) piling events in zoomed-out images of the floor area in both test cases. For feather scoring, the Segment Anything Model [24] was additionally used for individual bird segmentation, and YOLOv8 image classification models were trained to assign feather scores only to fully segmented hens, including the comb and without occlusions. Examples of the implemented camera views are presented in Section 3.3.

### 2.4. Data Visualisation

Results were analysed descriptively using frequencies, average ranking scores, and percentages. No inferential statistical analyses were performed. Results were visualised using JMP^®^ Pro software (version 17.0.0) and Python (version 3.9.18).

## 3. Results

### 3.1. Systematic Literature Review Outcomes

Figure 1 presents the PRISMA flowchart outlining the systematic literature review process.

**Figure 2:**
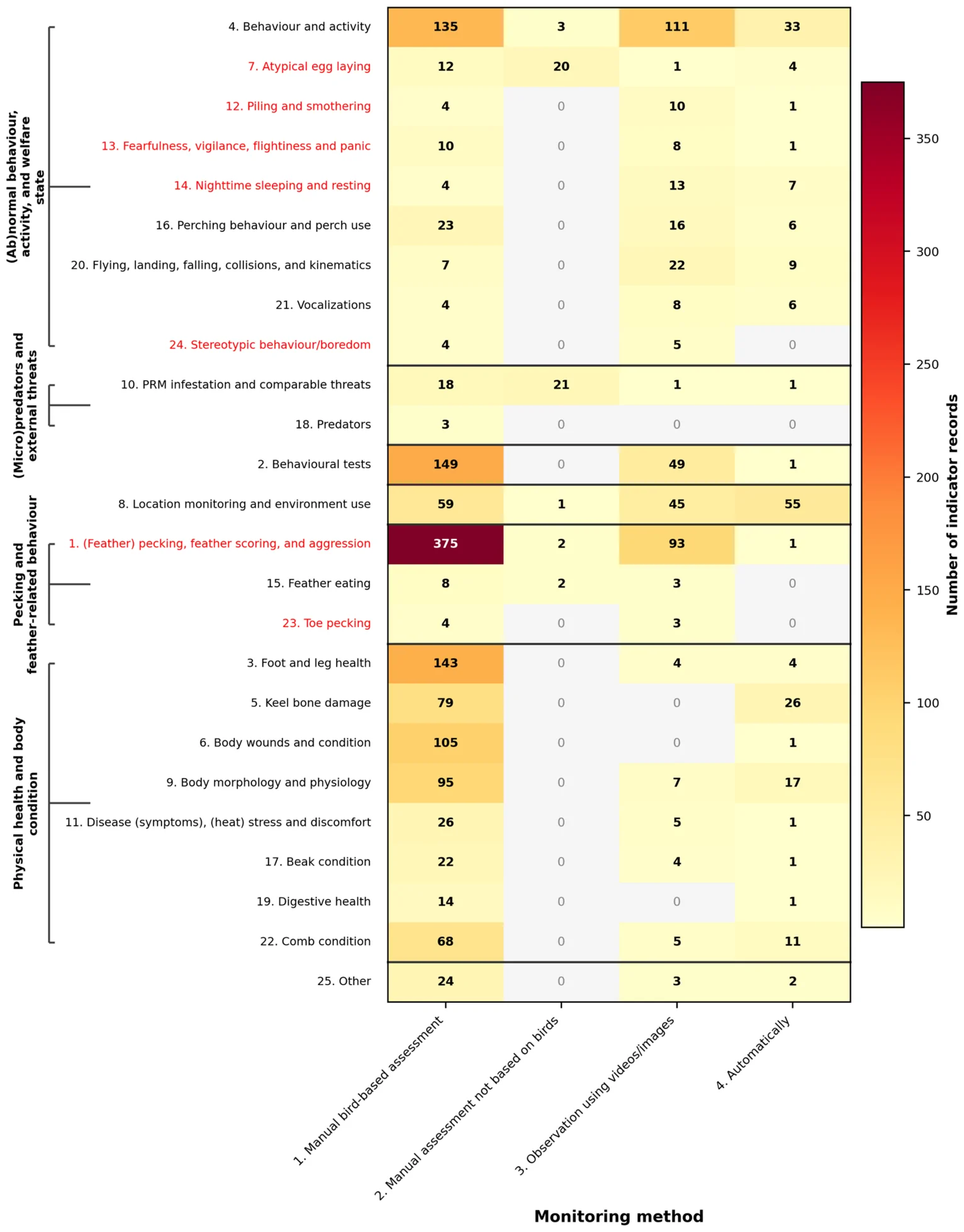
Frequency of monitoring methods (x-axis) used across the 25 welfare-related categories (y-axis) identified in the systematic literature review. Welfare-related categories are grouped into seven super-categories (displayed in bold on the y-axis; only four super-categories are displayed for readability). Welfare-related categories highlighted in red correspond to the seven unwanted behaviours discussed during the TREP meeting. Colour intensity represents the number of occurrences across the included articles. Full descriptions of all welfare-related categories are provided in Table 3.

Figure 2 presents the frequency with which each of the 25 welfare-related categories was investigated across the included articles, stratified by monitoring method. The seven welfare-related categories corresponding to the unwanted behaviours discussed during the TREP meeting are highlighted in red. Welfare categories were non-mutually exclusive, meaning that one article could contribute multiple observations.

(Feather) pecking, feather scoring, and aggression (category 1) were investigated most frequently, with 471 (375 + 2 + 93 + 1) occurrences. Behaviour and activity (category 4), behavioural tests (category 2), and foot and leg health (category 3) were also frequently investigated, with 282 (135 + 3 + 111 + 33), 199 (149 + 0 + 49 + 1), and 151 (143 + 0 + 4 + 4) occurrences, respectively. In contrast, the other welfare-related categories discussed during the TREP meeting were investigated considerably less frequently, including atypical egg laying (category 7; 37 occurrences), nighttime sleeping and resting (category 14; 24 occurrences), fearfulness, vigilance, flightiness and panic (category 13; 19 occurrences), piling and smothering (category 12; 15 occurrences), stereotypic behaviour and boredom (category 24; 9 occurrences), and toe-pecking (category 23; 7 occurrences).

Manual bird-based assessments were the predominant monitoring approach across the literature, often complemented by visual observations from images or videos. Automated monitoring was applied to most welfare-related categories, but generally at low frequencies. The highest numbers of automated measurements were observed for location monitoring and environment use (category 8; 55), behaviour and activity (category 4; 33), keel bone damage (category 5; 26), and body morphology and physiology (category 9; 17). Automated monitoring was particularly limited for the welfare-related categories discussed during the TREP meeting. (Feather) pecking, feather scoring, and aggression (category 1) had only one automated measurement, while nighttime sleeping and resting (category 14) and atypical egg laying (category 7) had seven and four, respectively.

### 3.2. TransRegional Expert Panel Responses

**Figure 3:**
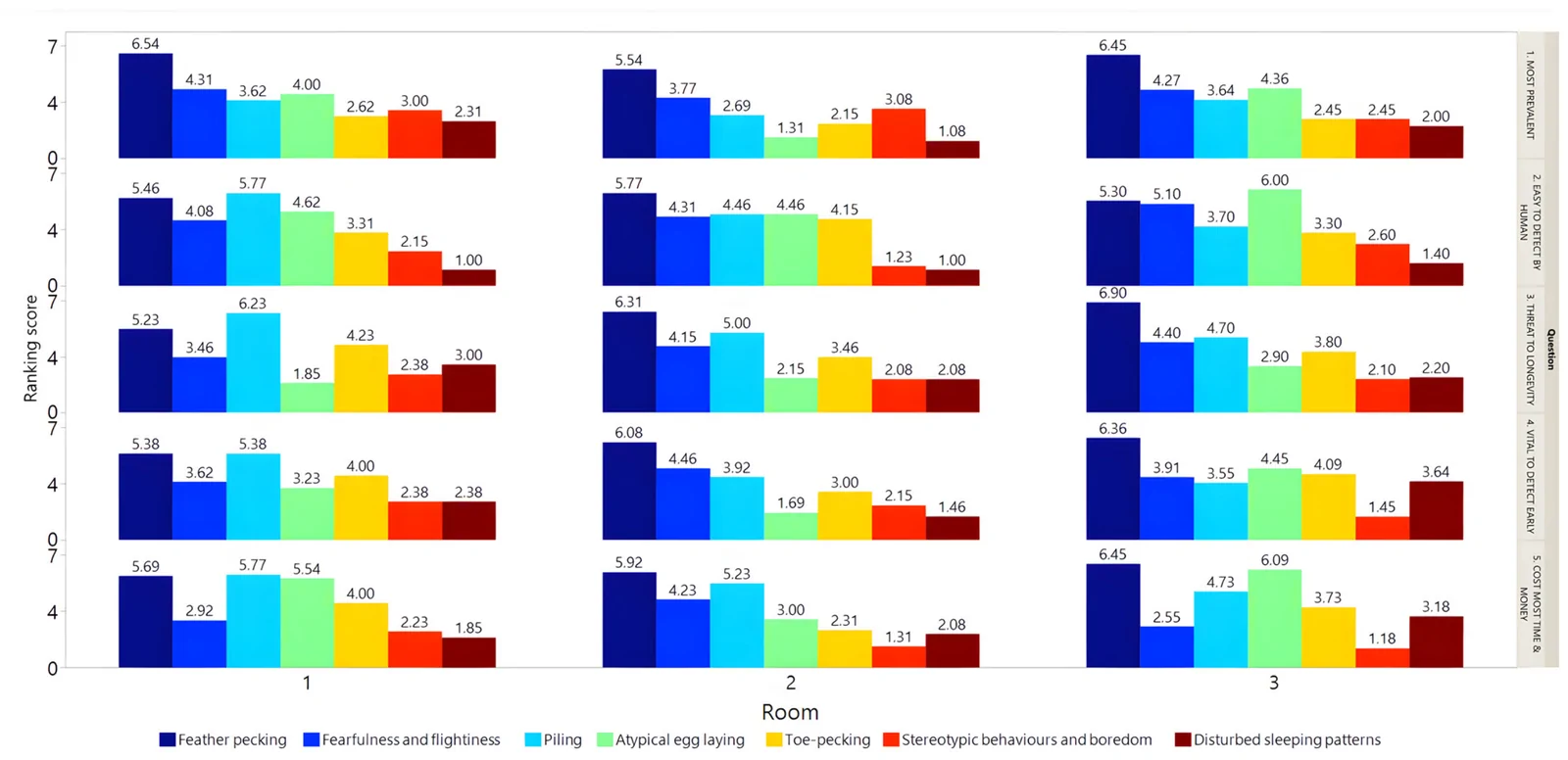
Aggregated TREP responses to the five ranking questions for Topic 1, presented separately for each of the three breakout rooms. Coloured bars represent the average ranking score (primary y-axis) within each room (x-axis) for the seven unwanted behaviours, with higher scores indicating higher rankings. The five panels (secondary y-axis) correspond to the five ranking questions listed in Table 4.

Figure 3 presents the aggregated breakout-room responses to the five ranking questions for Topic 1. Feather pecking was ranked highest across all five questions, with average ranking scores ranging from 5.51 to 6.18. Piling, as well as fearfulness and flightiness, also consistently appeared among the highest-ranked behaviours, although their relative importance differed between questions. Piling scored higher than fearfulness and flightiness for threat to longevity (5.31 versus 4.00), early detection (4.28 versus 4.00), economic cost (5.24 versus 3.23), and ease of human detection (4.64 versus 4.50). In contrast, fearfulness and flightiness scored higher for perceived prevalence (4.12 versus 3.32). Atypical egg laying scored particularly high for ease of human detection (5.02) and economic cost (4.88), whereas toe-pecking ranked relatively highly for threat to longevity (3.83), early detection (3.70), and economic cost (3.35). Stereotypic behaviour and boredom, together with disturbed sleeping patterns, consistently received average ranking scores below 3.

**Figure 4:**
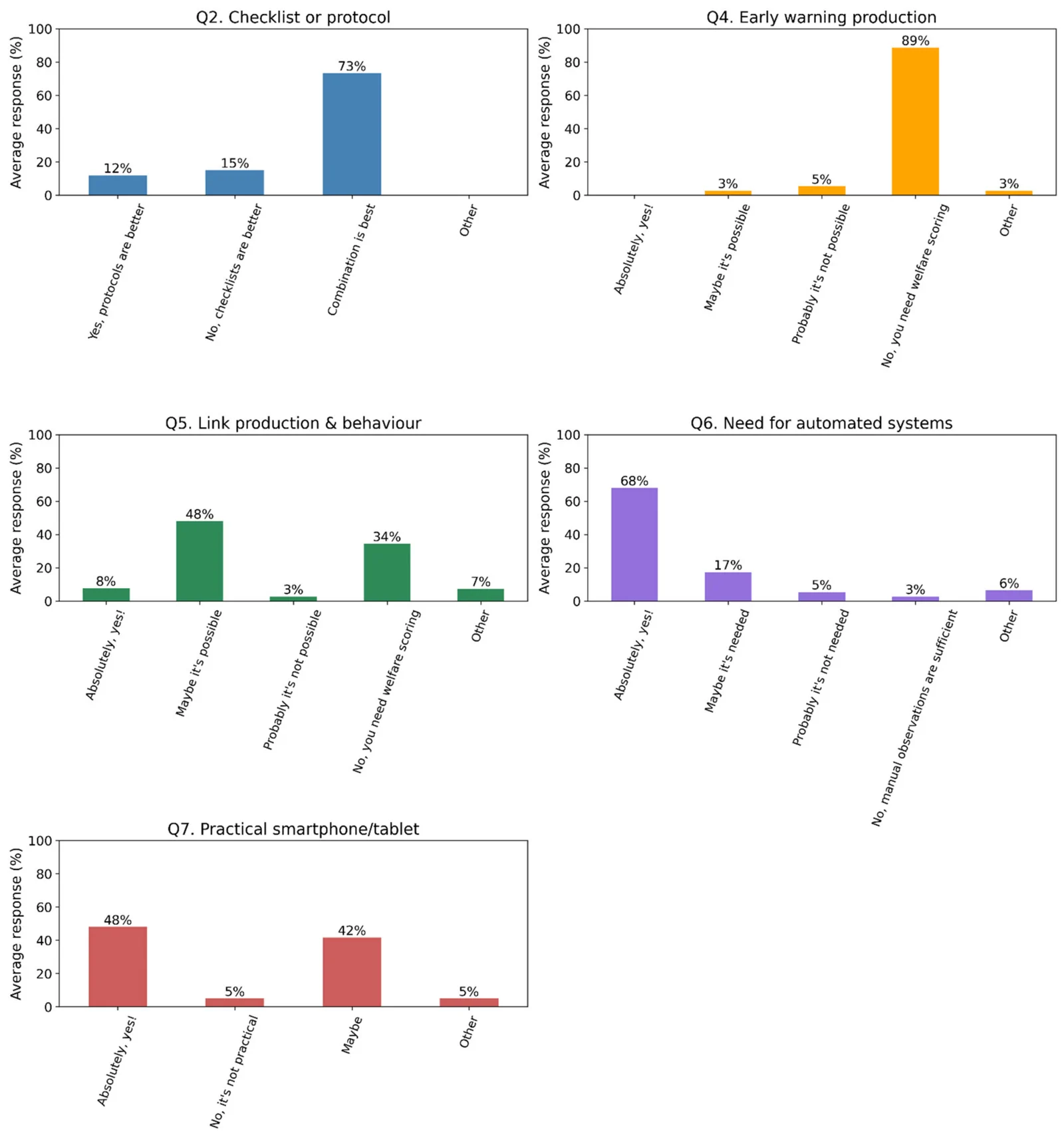
Aggregated TREP responses to the five multiple-choice questions for Topic 2, averaged across the three breakout rooms. Bars represent the average percentage (y-axis) for each response option (x-axis). The five subplots correspond to the five multiple-choice questions listed in Table 4.

Figure 4 presents the aggregated responses to the five multiple-choice questions for Topic 2, averaged across the three breakout rooms. Approximately 75% preferred combining time-intensive protocols with shorter checklists rather than relying exclusively on either approach. Nearly 90% indicated that production parameters alone were insufficient to generate reliable early warning signals and that additional welfare scoring was required. For the potential relationship between production parameters and unwanted behaviours, approximately 8% indicated that these could be directly linked, around half considered such a relationship possible, and approximately 35% indicated that additional welfare scoring would be required. Almost 70% indicated a clear need for objective scoring using automated monitoring systems, while nearly 20% considered such systems potentially necessary. Finally, approximately 50% considered the use of digital tools during barn walk-throughs to observe or score animals to be fully practical.

### 3.3. Proof-of-Concept: PTZ Camera Setup for Automated Monitoring in OMELETTE

Based on the priorities identified through the systematic literature review and TREP, the PTZ camera setups were configured to support monitoring of feather condition, floor or out-of-nest eggs, piling behaviour, and nighttime activity patterns using the same camera infrastructure.

**Figure 5:**
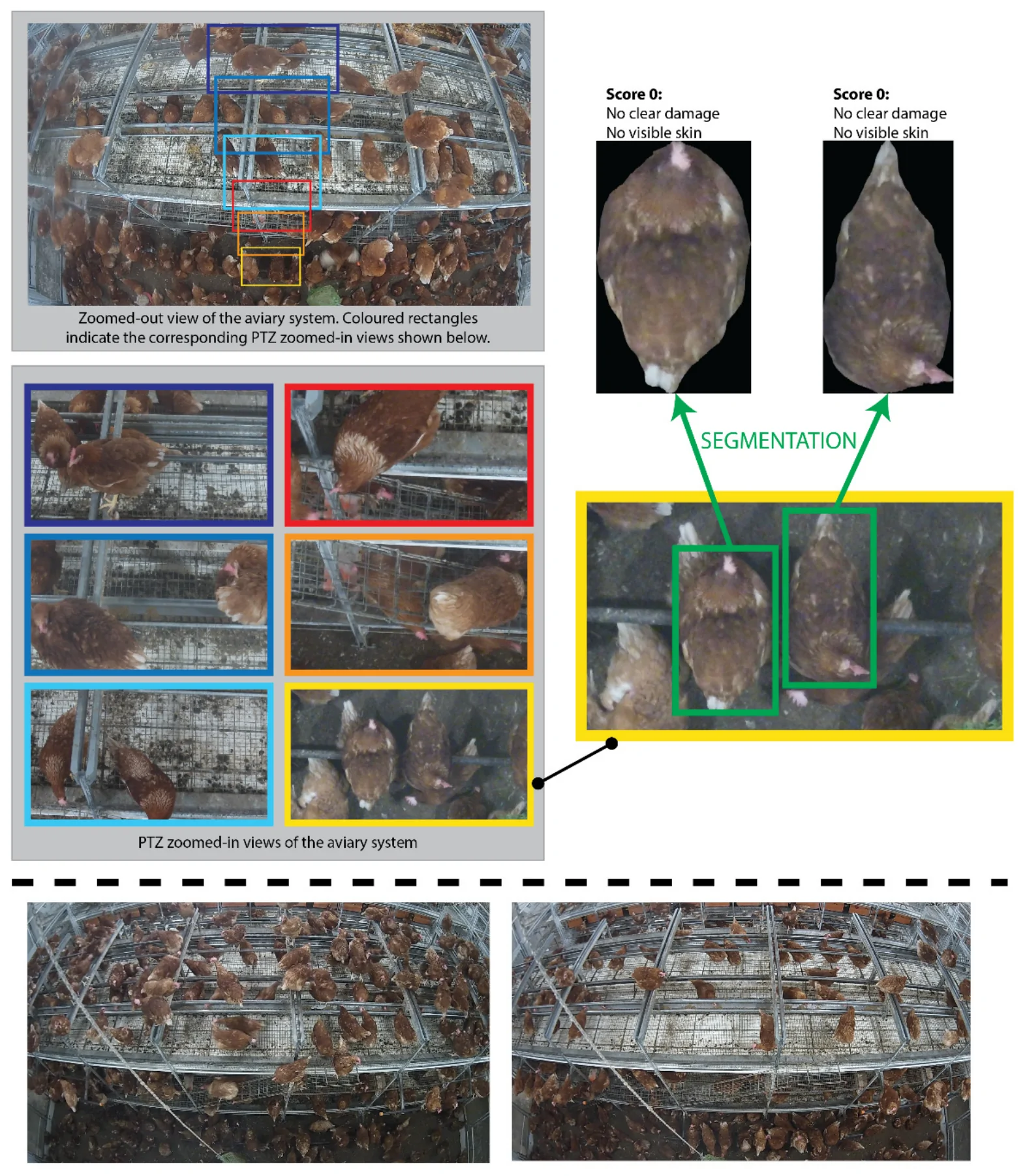
Example of PTZ camera presets used for individual hen detection and feather scoring in the first test case. The overview image (**top left**) shows the six zoomed-in aviary regions indicated by coloured rectangles, with the corresponding PTZ presets shown below. Green rectangles indicate detected hens within one selected preset. Images below the dashed black line compare hen presence immediately before lights-off (**left**; 17:00–18:00, see Section 2.3) with a daytime period (**right**). The greater hen presence before lights-off illustrates the importance of selecting appropriate monitoring periods for specific welfare challenges.

**Figure 6:**
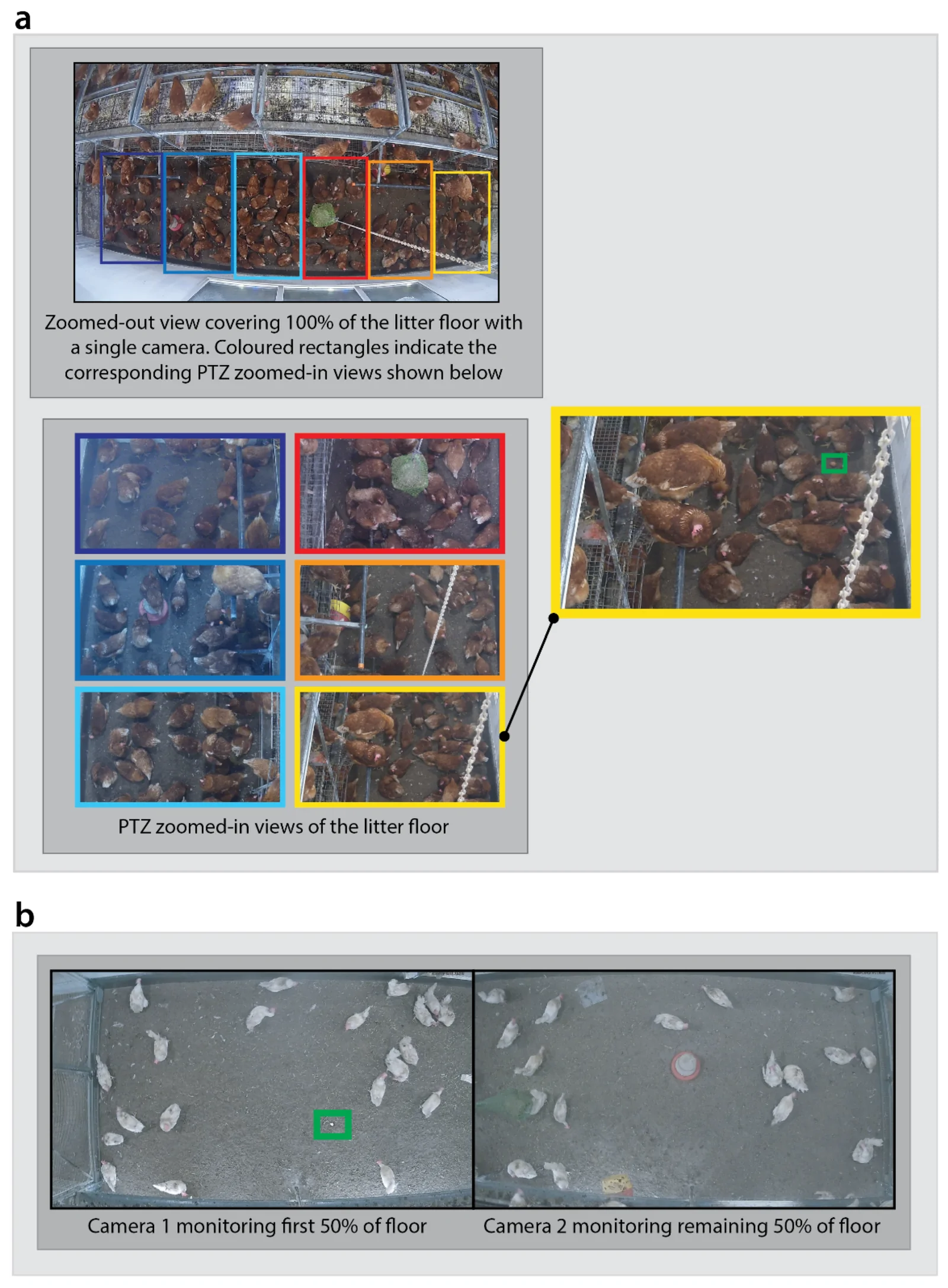
PTZ camera views used for floor or out-of-nest egg detection. (**a**) First test case, in which multiple zoomed-in PTZ presets were used to cover different sections of the litter area. (**b**) Second test case, in which greater camera coverage enabled floor egg detection from a wider field of view without requiring multiple zoomed-in litter presets. Green rectangles indicate detected floor eggs.

In the first test case, the two-camera configuration required multiple zoomed-in presets to obtain sufficiently detailed views of the aviary system for individual hen detection and feather scoring (Figure 5). Multiple zoomed-in presets were also required to cover different sections of the litter area for floor egg detection (Figure 6a). In contrast, the greater camera coverage in the second test case enabled floor egg detection from wider camera views without requiring multiple zoomed-in presets of the litter area (Figure 6b).

**Figure 7:**
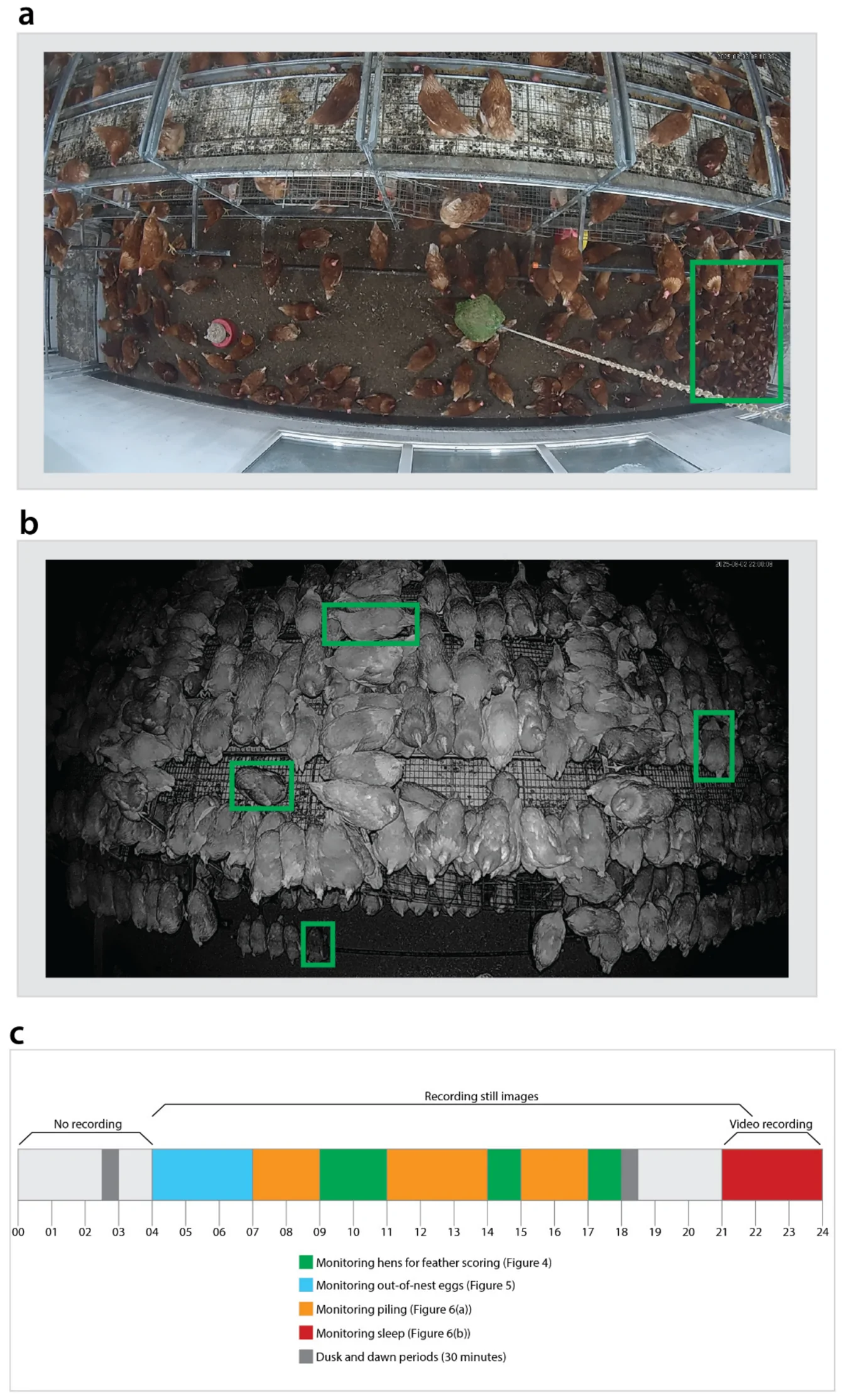
PTZ camera views and daily recording schedule used for integrated welfare monitoring. (**a**) Zoomed-out view of the litter area used for piling detection. (**b**) Zoomed-out view of the aviary system used for nighttime monitoring of individual hens. Green rectangles indicate detected piling events or individual hens. (**c**) Daily recording schedule applied in the first test case, showing the predefined periods allocated to floor egg monitoring, piling monitoring, feather scoring, and nighttime monitoring.

Zoomed-out camera views were used for welfare-monitoring tasks requiring a broader field of view. These included piling detection across the litter area (Figure 7a) and nighttime monitoring of individual hens across the aviary system (Figure 7b). Together, these camera views illustrate how predefined PTZ presets can be alternated throughout the day and night to support multiple monitoring tasks using the same camera infrastructure. The daily recording schedule applied in the first test case is illustrated in Figure 7c.

## 4. Discussion

Section 4.1 discusses the systematic literature review, while Section 4.2 compares the systematic literature review with the TREP outputs. Section 4.3 discusses the PTZ camera setup, while Section 4.4 provides an overview of future implications. Finally, Section 4.5 discusses the study limitations.

### 4.1. Systematic Literature Review Synthesis

In order to address the first objective of this work, the following paragraphs present the findings of the systematic literature review and provide answers to the three research questions formulated in the Introduction.

Figure 2 illustrates a wide variety of welfare-related categories which have been monitored or investigated. It highlights a strong imbalance in the distribution of welfare-related categories monitored in the literature on laying hens. Although a broad range of welfare indicators has been monitored in the literature between 2005 and 2025, (feather) pecking, feather scoring and aggression (category 1) clearly dominate the literature, while many other welfare-related categories have been monitored far less frequently. Indicators such as manual feather scoring and direct observation are well established, and feather pecking has become a central reference point for welfare assessment. In response to Research Question 1, this demonstrates that scientific attention has been concentrated on a limited subset of welfare challenges, particularly feather pecking, rather than being evenly distributed across the welfare spectrum. This aligns with previous work showing that feather pecking has been investigated over several decades and is framed as a key welfare challenge in the laying hen industry [25,26,27,28,29,30]. Other welfare challenges have gained attention more recently, including piling behaviour [31] and disrupted nighttime sleep [23,32]. One particularly persistent and highly prevalent welfare challenge is infestation with PRM, a nocturnal blood-feeding ectoparasite associated with irritation, restlessness at night, anaemia, reduced egg quality, and increased mortality in severe infestations [33]. Structural changes associated with the transition towards cage-free systems have inadvertently increased opportunities for PRM proliferation by creating additional refuges and environmental complexity, complicating effective control [34]. Within this context, monitoring nighttime activity and perch occupation of laying hens might provide additional insight into the presence and proliferation of PRM [23]. Despite its major impact, only a limited number of studies were found to monitor PRM. The seven unwanted behaviours presented at the interactive TREP meeting, with the exception of feather pecking, received comparatively less research attention. Among these behaviours, atypical egg laying was monitored most frequently, primarily through the manual collection of floor eggs. Other welfare challenges, such as toe-pecking [35], have received even less research attention.

With respect to monitoring methods shown in Figure 2, addressing Research Question 2, the literature remains heavily dominated by manual bird-based assessments, occasionally complemented by video or image observations. Fully automated measurements have been applied far less often, indicating that welfare monitoring to date largely relies on human observation rather than objective, technology-driven approaches. Only four of the 25 welfare-related categories, namely location monitoring and environment use (category 8), behaviour and activity (category 4), keel bone damage (category 5) and body morphology and physiology (category 9), received relatively greater attention in terms of automated monitoring, with location monitoring and environment use (category 8) being monitored most frequently using automated techniques. Consequently, many other welfare-related categories remain comparatively underexplored from an automated monitoring perspective, highlighting substantial untapped potential for precision livestock farming applications. These findings are consistent with the review of PLF technologies in the poultry sector by [3], who reported that the vast majority of identified systems were still at a prototype stage, with only a small fraction representing commercially available monitoring solutions. Most PLF studies relied on sensor-based approaches, with cameras being more commonly used than acoustic systems. Their review further showed that PLF research has focused predominantly on broiler production, with comparatively fewer applications developed for laying hens. Although animal health and welfare were frequently cited objectives, welfare-focused systems primarily relied on behavioural measurements, most often locomotor activity, highlighting both the narrow scope of automated welfare indicators currently addressed and the substantial potential for broader, technology-driven welfare monitoring.

Taken together, these findings directly address Research Question 3 by demonstrating that the application of automated monitoring tools across welfare-related categories remains low. While feather pecking is relatively well characterised and routinely monitored or measured in the literature, substantial gaps persist for many other welfare-related categories. This imbalance underscores clear opportunities for future research to broaden the scope of welfare monitoring beyond feather pecking by addressing underrepresented welfare challenges and expanding automated and objective PLF-based assessment.

### 4.2. Integration of Systematic Literature Review and TREP Outcomes

To address the second objective of this study, the findings of the systematic literature review were compared with the priorities and perspectives expressed during the TREP meeting. This comparison provides insight into the extent to which current scientific attention aligns with sector priorities, particularly for the seven unwanted behaviours discussed during the TREP, and whether current monitoring approaches correspond to stakeholder needs.

#### 4.2.1. Seven Unwanted Behaviours

The strongest alignment between the systematic literature review and the TREP was observed for feather pecking. Feather pecking was the most extensively monitored welfare challenge in the literature, with 471 occurrences (see Figure 2), and was consistently prioritised across all five TREP ranking dimensions (see Figure 3), reinforcing its established position as a major welfare and economic concern in the laying hen industry [27,36,37]. This prioritisation is further supported by commercial farm data. Ref. [38] found signs of severe feather damage in 49% of the laying flocks investigated, illustrating the high prevalence of the problem under commercial conditions. In addition, ref. [39] reported cumulative mortality ranging from 0.5 to 9.0% across 39 commercial Norwegian flocks, with higher mortality associated with greater feather loss, which the authors suggested could potentially reflect severe feather pecking and subsequent cannibalism. Together, the literature review, TREP rankings, and commercial flock data demonstrate a strong convergence between scientific attention and perceived sector importance for feather pecking.

In contrast, piling and smothering showed a clear discrepancy between research attention and stakeholder prioritisation. Piling was prioritised particularly in relation to threat to longevity, early detection, and economic consequences, yet was identified only 15 times in the reviewed literature, with only one occurrence involving automated monitoring. Its high stakeholder ranking is consistent with commercial farm observations. In UK free-range flocks, nearly 60% of farm managers reported smothering in their previous flock, with an average loss of 25.5 hens per smothering event [40]. These findings indicate that, although piling receives comparatively limited research attention and may be less prevalent than some other unwanted behaviours, individual events can have severe consequences for hen longevity and farm productivity. This discrepancy suggests that current research and technology development may not yet adequately reflect its perceived on-farm importance.

Fearfulness and flightiness showed a similar imbalance between stakeholder perceptions and research attention. Experts perceived these behaviours as relatively prevalent and comparatively easy to detect during routine farm inspections, yet they were identified only 19 times in the literature, with one occurrence involving automated monitoring. In contrast to piling, however, fearfulness and flightiness ranked lower in relation to longevity and economic consequences. This distinction illustrates that the prevalence of a welfare challenge does not necessarily correspond to the severity of its consequences and may explain why stakeholder priorities differed across the five ranking dimensions.

Atypical egg laying was considered particularly relevant because of its ease of detection and economic implications, while remaining comparatively underrepresented in the literature, with 37 occurrences, of which four involved automated monitoring. This perceived economic relevance is consistent with observations from 69 commercial Australian brown egg-laying flocks, where floor eggs were identified as an economic cost because they require manual collection and are downgraded [41]. Together, these findings indicate an opportunity for further development of monitoring approaches targeting a welfare-related challenge with direct practical and economic relevance to commercial production.

Toe-pecking was mainly prioritised in relation to longevity and early detection, despite its comparatively low perceived prevalence, and was among the least investigated welfare challenges in the literature, with only seven occurrences and no automated monitoring identified. This pattern is consistent with field observations indicating that toe-pecking may occur less frequently but can have severe consequences when outbreaks develop. A Swiss survey identified toe-pecking as a cause of substantial mortality and economic loss and reported a greater occurrence in white compared with brown laying hens [35]. More recently, a longitudinal study of commercial Danish flocks demonstrated substantial variation between flocks, with toe-pecking accounting for 46% of diagnosed mortality in one affected barn flock [42]. The comparatively high ranking of toe-pecking for early detection and longevity may therefore reflect the potential severity of outbreaks rather than its overall prevalence. Its limited representation in the literature, particularly with respect to automated monitoring, suggests that low research attention should not necessarily be interpreted as evidence of limited welfare relevance.

Disturbed sleeping patterns represented a different type of discrepancy. Although disturbed sleep consistently received low TREP ranking scores, the existing literature emphasises the importance of sleep for animal welfare and suggests that nighttime conditions are often overlooked in welfare assessment [32]. Nighttime sleeping and resting were identified only 24 times in the review, with seven occurrences involving automated monitoring. The relatively low representation of nighttime welfare in both the literature and stakeholder rankings may therefore reflect, at least partly, the practical difficulty of observing hens during dark periods rather than an absence of welfare relevance.

Stereotypic behaviour and boredom were underrepresented, with nine occurrences in the reviewed literature, and also received comparatively low priority during the TREP. This convergence suggests that these behaviours currently receive relatively limited attention in both research and practice. However, as with disturbed sleep, difficulties in recognising these behaviours during routine farm inspections may contribute to their lower perceived priority.

Overall, a comparison of the systematic literature review, TREP rankings, and available commercial farm data reveals both convergence and important gaps between scientific attention and sector priorities. Feather pecking shows strong alignment, being both extensively investigated and consistently prioritised by stakeholders. In contrast, piling and toe-pecking illustrate how welfare challenges receiving comparatively little research attention can nevertheless have severe consequences when they occur. More prevalent welfare challenges are therefore not necessarily those associated with the greatest acute mortality or production losses. Prioritisation of future welfare monitoring should consequently consider not only research frequency and perceived prevalence, but also the magnitude and immediacy of potential welfare and economic consequences and the extent to which early detection could prevent escalation.

#### 4.2.2. Monitoring Approaches and Opportunities for Integrated Welfare Monitoring

The TREP responses concerning monitoring approaches further complement the findings of the systematic literature review. The review demonstrated that welfare monitoring in laying hens remains strongly dominated by manual bird-based assessments, while automated monitoring has been applied comparatively infrequently across most welfare-related categories (see Figure 2). This contrasts with the clear stakeholder interest in more objective monitoring approaches. Almost 70% of TREP participants indicated a clear need for objective scoring using automated monitoring systems, while a further proportion considered such systems potentially necessary (see Figure 4). This suggests that current technology development and application may not yet fully meet the perceived needs of the sector.

At the same time, the TREP responses did not indicate that automated monitoring should replace existing manual welfare assessment. Approximately 75% of participants preferred combining more comprehensive and time-intensive welfare assessments with shorter checklists rather than relying exclusively on either approach. Similarly, nearly 90% considered production parameters alone insufficient for generating reliable early warning signals and indicated that additional welfare scoring was required. These findings support an integrated monitoring strategy in which automated measurements, direct welfare assessments, and production information provide complementary information rather than functioning as isolated sources of evidence.

This interpretation is consistent with the wider distribution of monitoring approaches identified in the literature. Although automated monitoring remained limited overall, comparatively greater use was observed for location monitoring and environmental use, behaviour and activity, keel bone damage, and body morphology and physiology. Behaviour and activity alone accounted for 282 occurrences in the literature, including 33 applications of automated monitoring, supporting the broader observation that behavioural measures are frequently targeted by PLF technologies [3]. Behavioural testing and foot and leg health also received substantial research attention, with 199 and 151 occurrences, respectively, although automated applications remained comparatively limited. These findings indicate that existing research provides a broad base of welfare-related measurements, but that their integration into automated and practically applicable monitoring systems remains underdeveloped.

The TREP responses also suggest that digital technologies could be incorporated into existing management routines rather than requiring entirely new assessment procedures. Approximately half of the participants considered the use of tablets or smartphones while observing or scoring hens during barn walk-throughs to be fully practical. Such tools could therefore provide a practical interface through which manual observations, production information, and automated measurements are combined and interpreted within the same monitoring framework.

Taken together, the literature review and TREP outcomes indicate a need to move beyond both purely manual welfare assessment and isolated automated measurements. The literature demonstrates that many welfare-related categories remain insufficiently addressed by automated approaches, while the TREP highlights stakeholder demand for objective monitoring without abandoning established manual assessments. Integrating these complementary sources of information may therefore provide a more realistic pathway towards continuous and practically relevant welfare monitoring. This combined evidence also provides the rationale for the multipurpose PTZ camera approach presented in the following section, in which several welfare challenges identified through both scientific evidence and stakeholder input are targeted using the same camera infrastructure.

### 4.3. Proof-of-Concept: PTZ Camera Setup for Automated Monitoring in OMELETTE

To address the third objective of this study, the PTZ proof-of-concept demonstrates the potential of adapting the camera field of view and recording schedules to monitor different welfare challenges using the same camera infrastructure. Rather than requiring separate monitoring systems, PTZ cameras could therefore provide a multipurpose platform in which camera settings are adapted according to the welfare challenge being assessed.

The comparison between the two test cases highlights an important trade-off between camera number and visual coverage. Reducing the number of cameras may lower hardware requirements, but it increases the importance of strategic camera positioning, zoom levels, predefined PTZ presets, and recording schedules to achieve sufficient coverage. Conversely, greater camera coverage allows wider fields of view to be used and can reduce the need for multiple predefined presets.

A similar trade-off applies to the type of data collected. Daytime still images reduce data storage and processing requirements and enable repeated monitoring throughout the production day, but are less suitable for analysing dynamic behaviours that require continuous temporal information. Video recordings provide richer behavioural information but substantially increase data volume and processing requirements. Future camera-based welfare-monitoring systems should therefore balance spatial coverage, temporal resolution, data requirements, and the specific welfare challenges being monitored.

The proof-of-concept also demonstrates that the timing of monitoring should be adapted to the welfare challenge. Figure 5, for example, shows that monitoring feather coverage shortly before lights-off is advantageous because more birds are positioned on the aviary system and closer to the cameras. As reflected in the recording schedule in Figure 7c, strategically allocating monitoring periods may therefore improve both visibility and monitoring efficiency.

Overall, the proof-of-concept supports the third objective by showing how welfare priorities identified through the literature review and TREP can be translated into an integrated PTZ-based monitoring framework. At the same time, it highlights key design considerations for future commercial implementation, including camera coverage, camera positioning, temporal resolution, data requirements, and welfare-specific monitoring schedules.

### 4.4. Implications for Future Research and Development

Building on the gaps identified in the literature, the priorities expressed during the TREP, and the PTZ proof-of-concept, several directions emerge for the future development and implementation of integrated welfare monitoring in commercially housed laying hens.

#### 4.4.1. Cost-Efficient and Robust Camera-Based Hardware Configurations

Future research should explore cost-efficient hardware configurations that enable sufficient coverage of large and structurally complex housing systems without requiring a proportional increase in camera numbers. One promising approach is to combine PTZ functionality with camera railing or mobile mounting systems, allowing a limited number of cameras to cover multiple locations while dynamically adjusting zoom level and viewing angle. Such configurations may reduce hardware and maintenance requirements while preserving the ability to monitor multiple welfare challenges across different functional areas of the laying house. This is particularly relevant given farmers’ concerns regarding maintenance, unproven benefits, pricing, data ownership, data access, and rural internet connectivity [6,7]. Although camera rail-based systems have already been proposed [43], their technical and economic added value under commercial conditions remains to be demonstrated. Future applied research should therefore evaluate whether these configurations provide measurable benefits in terms of welfare monitoring, hen longevity, productivity, and practical value for end users.

#### 4.4.2. From Automated Measurements to Actionable Insights Through PDCA and Digital SOPs

Future PLF development should move beyond automated measurement and focus on translating monitoring outputs into clear and actionable management decisions. This need is supported by persistent uncertainty regarding return on investment and limited practical evidence showing how PLF outputs can be embedded into routine farm management [4,5,13,14]. Moreover, the complex and farm-specific nature of laying hen housing systems makes universal monitoring schemes or intervention protocols unlikely to be effective across all contexts [7,17]. Decision-support systems should therefore provide interpretable, context-specific feedback that enables farmers to respond appropriately to emerging welfare issues.

Embedding such outputs within a Plan–Do–Check–Act (PDCA) framework [18] could support continuous and adaptive flock management. For example, elevated nighttime activity detected by cameras at a specific perch location could trigger intensified PRM monitoring using cardboard traps or visual scoring (Plan), followed by a targeted intervention such as the release of predatory mites (Do). Subsequent changes in camera-derived activity patterns and trap or visual measurements could then be evaluated (Check), after which the intervention could be continued, adjusted, terminated, or supplemented with additional local treatments such as silica-based products (Act). This illustrative example demonstrates how automated measurements, manual monitoring, and targeted interventions could be combined within digital standard operating procedures (SOPs).

PDCA-based monitoring may also facilitate applied research under semi-commercial and commercial conditions, where conventional controlled experiments can be difficult to implement over long production cycles. Repeated, small-scale interventions within ongoing production cycles could instead be evaluated using both automated and manual measurements. Such iterative evaluations may generate practical evidence on the effectiveness of specific SOPs while supporting their gradual refinement under commercial conditions.

Future research should therefore focus on developing and validating farm-specific digital SOPs that incorporate automated monitoring within existing management and advisory workflows. These tools may support not only farmers, but also veterinarians, advisors, and other professionals involved in flock management.

#### 4.4.3. Integrating Heterogeneous Data Streams for Holistic Welfare Management

Future decision-support systems should integrate complementary data streams, including production metrics, manual welfare assessments, automated camera-based measurements, and environmental data. This partly aligns with [44], who advocate a shift from subjective, labour-intensive and unimodal welfare assessment towards data-driven, multimodal monitoring integrating visual, acoustic, environmental, and physiological information. However, whereas [44] emphasise moving away from traditional manual welfare assessment, the TREP outcomes suggest that manual and automated approaches should remain complementary rather than one replacing the other. Because laying hen welfare is influenced by interacting behavioural, environmental, and management factors, isolated indicators are unlikely to provide sufficient information for effective decision-making.

PTZ cameras could contribute multiple welfare-related measurements within such an integrated framework rather than targeting individual outcomes in isolation. Future work should therefore broaden automated monitoring beyond well-studied challenges such as feather pecking and validate emerging indicators under commercial conditions.

#### 4.4.4. PTZ Recording Schemes as Reusable Monitoring Templates

The recording schedule presented in Figure 7c provides an initial example of how PTZ presets can be allocated to different welfare-monitoring tasks throughout the day. The same approach could be extended to additional welfare indicators or adapted to use video rather than still images when continuous behavioural information is required.

Future studies should investigate whether standardised PTZ recording schemes can be developed around lighting schedules, housing layout, management routines, and hen activity patterns while retaining sufficient flexibility for farm-specific adaptation. Such templates could improve consistency in data collection and facilitate the implementation of integrated welfare monitoring across different commercial settings.

### 4.5. Study Limitations

Several limitations of this study should be acknowledged.

First, welfare challenges were grouped into predefined categories to enable comparison across studies that used heterogeneous terminology. While this categorisation was necessary for synthesis, it may have resulted in the loss of some nuance between closely related indicators or study-specific definitions of welfare outcomes.

Second, the TREP outcomes reflect expert perceptions at a specific point in time and within a specific geographical and sectoral context. Although the panel included a broad range of stakeholder groups and participants from several European countries, the geographical distribution was uneven, with Belgian participants representing the largest group among participants for whom country information was available. This geographical imbalance may have influenced the prioritisation of welfare challenges and perceptions of monitoring approaches, as production systems, welfare practices, and sector priorities may differ between countries. The TREP outcomes should therefore not be interpreted as representative of the entire NWE laying hen sector.

Third, the PTZ camera setup presented in this study is intended as a conceptual proof-of-concept rather than a validated monitoring system. No empirical performance metrics, validation against ground truth, or economic assessments were conducted. As such, the feasibility, robustness, and cost–benefit balance of the proposed configurations under diverse commercial conditions remain to be evaluated in future applied studies.

Finally, the camera-based examples focus on selected welfare challenges identified as priorities through the literature review and TREP. Other relevant welfare issues, including those perceived as lower priority or more difficult to monitor automatically, were not addressed and warrant further investigation as monitoring technologies and analytical methods continue to evolve.

## 5. Conclusions

Taken together, the literature review, expert panel outcomes, and proof-of-concept examples presented in this study highlight the need for future welfare-monitoring approaches in commercially housed laying hens to move beyond isolated measurements and small-scale trials towards more integrated, cost-effective, and farm-specific systems. Such systems should be capable of supporting adaptive, data-informed, PDCA-like management strategies under commercial conditions through the use of digital SOPs. Within this context, PTZ cameras, potentially combined with railing systems, could support the generation of actionable insights by enabling automated, scheduled, and location-specific data collection through preconfigured recording schemes and PTZ presets. The systematic literature review revealed a clear imbalance in research attention across welfare-related categories in laying hens. Feather pecking dominates the literature, whereas several other welfare challenges remain comparatively underrepresented. While long-standing issues such as feather pecking show strong convergence between published research and expert opinion, other challenges highlight gaps where research activity does not yet fully reflect perceived on-farm relevance. The outcomes of the TREP further underline the need for integrated welfare-monitoring approaches that combine time-intensive assessments, shorter checklists, and automated systems, rather than relying on single indicators or methods. Experts also considered the use of digital devices, such as cameras, tablets, or smartphones, during routine barn walk-throughs to be practically feasible, supporting their integration into daily farm management.

In addition, this study illustrates how insights from both the literature review and the TREP can be translated into practical monitoring solutions using PTZ camera technology. The proof-of-concept implemented within the OMELETTE project shows how welfare challenges identified as both relevant and feasible can be operationalised through a single, multipurpose camera system. By targeting priority issues such as feather pecking, piling, and floor egg laying, alongside less frequently monitored indicators such as sleep, the PTZ setups demonstrate how integrated camera-based monitoring can move beyond isolated measurements towards a more holistic assessment of flock welfare. By addressing its three main objectives, this study provides an integrated overview of the current state of welfare monitoring in laying hens and identifies key directions for future computer-vision-based PLF research, development and adoption.

## Figures and Tables

**Table 1:** Welfare-related concepts included in Search 1, corresponding search terms, and rationale for inclusion.

Welfare Concept	Search Terms	Rationale for Inclusion
Egg eating and destruction	“Egg eat*”; “Egg destruct*”	Included to capture undesirable egg directed behaviours that may affect hen welfare and production.
Feather pecking and feather damage	“Feather peck*”; “Feather pick*”; “Feather loss”; “Feather damage”; “Plumage loss”; “Plumage damage*”	Included because feather pecking and resulting plumage damage are important and widely recognised welfare problems in laying hens.
Fearfulness and flightiness	Fearfulness; Flightiness	Included to capture fear related behavioural responses that may reflect negative welfare states and affect flock management.
Piling and smothering	Piling; Smothering	Included because piling can result in injury and mortality through smothering and represents an important flock welfare risk.
Stereotypic behaviour and boredom	“Stereotypic behaviour*”; “Stereotypic behavior*”; Boredom	Included to capture repetitive or abnormal behaviours potentially associated with behavioural restriction, insufficient stimulation, or unsuitable housing conditions.
Toe pecking	“Toe-peck*”; “Toe peck*”; “Toe pick*”	Included because toe pecking is a damaging behaviour that can cause wounds, pain, and subsequent health problems.
Keel bone damage	“Keel bone deform*”; “Keel bone damage”; “Keel bone fracture*”	Included because keel bone fractures and deformities are important physical welfare problems in laying hens and may cause pain and impaired mobility.
Mortality	“Dead bird*”; Mortalit*	Included because mortality is an important flock level outcome that may reflect underlying health, welfare, or management problems.
Atypical egg laying	“Floor egg*”; “Out-of-nest egg*”; “Out of nest egg*”; “Atypical egg laying”	Included to capture laying outside designated nest areas, which may reflect behavioural, housing, or management related problems and has practical and economic consequences.
Disturbed nighttime sleep	“Disturbed sleep*”; “Sleep disrupt*”	Included because disturbed nighttime resting and sleeping patterns may indicate environmental disturbance, stress, disease, or other welfare challenges.
Respiratory problems	“Respiratory problem*”; “Breathing problem*”; “Respiratory disease*”	Included because abnormal breathing and respiratory disease are direct indicators of impaired health and welfare.
Eye problems	“Eye problem*”; “Eye irritation”	Included because eye abnormalities or irritation may indicate disease, injury, or adverse environmental conditions.
Aberrant vocalisations	“Aberrant vocali*”; “Pain squawk”; “Distress call*”	Included because changes in vocalisation, including pain and distress calls, may provide behavioural indicators of negative welfare states.
Foot injuries and wounds	“Foot injur*”; “Foot wound*”	Included because foot injuries can cause pain, impaired locomotion, and reduced welfare.
Body wounds and lesions	“Body wound*”; “Body lesion*”; “Integument damage*”; “Integument lesion*”	Included because wounds and integument lesions are direct indicators of physical damage and compromised welfare.
Head wounds and lesions	“Head wound*”; “Head lesion*”	Included to capture physical injuries to the head as indicators of damaging interactions or other welfare problems.
Footpad lesions and dermatitis	“Footpad lesion*”; “Foot pad lesion*”; “Footpad dermatitis”; “Foot pad dermatitis”; Bumblefoot; Pododermatitis	Included because footpad lesions and inflammatory foot conditions can cause pain, impaired locomotion, and reduced welfare.
Poultry red mite infestation	“Poultry red mite”; “Dermanyssus gallinae”; PRM	Included because poultry red mite infestation is an important laying hen welfare problem associated with irritation, disturbed nighttime behaviour, stress, anaemia, and potentially increased mortality.

* Wildcard to capture variation in root words.

**Table 2:** Automated monitoring concepts included in Search 2, corresponding search terms, and rationale for inclusion.

Automated Monitoring Concept	Search Terms	Rationale for Inclusion
Acoustic and vocal monitoring	“Acoustic monitoring”; “Audio signal processing”; “Noise analysis”; “Sound analysis”; “Vocali?ation analysis”	Included to capture automated approaches that use flock sounds or vocalisations as signals for behaviour, health, or welfare monitoring.
General automated and real-time monitoring	“Automated monitoring”; “Automated welfare”; “Real-time monitoring”	Included as broad terms describing automated or continuous systems developed to monitor animal welfare or related farm outcomes.
Precision and smart farming	PLF; “Precision livestock farming”; “Precision agriculture”; “Intelligent farming”; “Smart farming”	Included to capture studies framed within precision or smart farming approaches that use technology and data to support automated livestock monitoring and management.
Data-driven monitoring and analytics	“Big data”; “Control chart”; “Signal analysis”	Included to capture data-processing and analytical approaches used to automatically identify patterns, deviations, or changes in animal or farm measurements.
Sensors and biosensors	Biosensor; Sensor; Wireless	Included to capture sensor-based and wireless systems used for automated collection and transmission of animal, environmental, or production data.
RFID and ultra-wideband tracking	RFID; UWB	Included to capture automated identification, localisation, movement, and resource-use monitoring technologies commonly applied at individual-animal level.
Computer vision and image-based monitoring	“Image analysis”; “Machine vision”; “Optical flow”	Included to capture automated image- and video-based approaches for measuring animal behaviour, activity, distribution, or physical characteristics.
Infrared thermal imaging	“Infrared thermal imaging”; “Infrared thermal image”; “Infrared thermography”	Included to capture non-contact thermal imaging approaches used to automatically assess physiological or health-related changes.
Integrated management systems	“Integrated management system”	Included to capture systems that combine multiple data streams or monitoring technologies within an integrated farm-management framework.
Precision feeding and nutrition	“Precision feeding”; “Precision nutrition”	Included to capture automated approaches that use individual- or flock-level data to optimise feeding or nutritional management.
Optical transmission measurements	“Transmission color value”; “Transmission colour value”	Included to capture automated optical measurement approaches represented in the adapted PLF search strategy.

**Table 3:** Overview of the 25 welfare-related categories and the corresponding super-category.

Super-Category	Welfare-Related Category and Category Number	Examples of Terminology Extracted from the Included Articles, Using the Authors’ Original Wording
Pecking and feather-related behaviour	1. (Feather) pecking, feather scoring, and aggression	Welfare challenges related to feather pecking, object pecking, feather scoring and aggression. Ground and litter pecking were considered foraging and were classified into behaviour and activity (category 4).
	15. Feather eating	Welfare challenges related to feather eating and tests on feathers eaten.
	23. Toe-pecking	Welfare challenges related to toe-pecking behaviour.
Assessment methods	2. Behavioural tests	Welfare challenges related to using behavioural tests to measure fear, avoidance, cognition, visual discrimination, choices, isolation and emotion. Any tests measuring fear are classified in this category and not into fearfulness, vigilance, flightiness and panic (category 13).
Physical health and body condition	3. Foot and leg health	Welfare challenges related to foot and leg measurements, including footpad dermatitis, bumblefoot, claw/nail length, wounds on the feet, feet/toe damage, (footpad) temperature, gait score, lameness, asymmetry of feet/legs.
	5. Keel bone damage	Welfare challenges related to keel bone.
	6. Body wounds and condition	Welfare challenges related to skin lesions, body wounds on head/neck/wattle/back/tail/wing/cloaca/breast/belly/vent/rear body/rump, integument for skin scoring, skin injuries from cannibalism, and dermatitis. Excluding lesions or wounds for foot and leg (category 3), keel (category 5), beak (category 17) and comb (category 22).
	9. Body morphology and physiology	Welfare challenges related to body weight, heart and respiration rate, limb dimensions, temperature of body/breast/rectum/eye/earlobe/face/wattle/wing/shank, asymmetry and length of wings and wattle. Excluding measurements for foot and leg (category 3), keel (category 5), beak (category 17) and comb (category 22).
	11. Disease (symptoms), (heat) stress and discomfort	Welfare challenges related to panting, abnormal breathing, disarranged feathers, drooped wings, dull eyes, disease signs and non-characteristic behaviour, eye abnormalities, restless behaviour, visibly ill.
	17. Beak condition	Welfare challenges related to beak condition/damage/length/temperature/colour/deformities/lesions/morphology/shape/sensitivity tests/fissures.
	19. Digestive health	Welfare challenges related to enlarged crop, abnormal manure, cloacal discharge/condition, diarrhoea, swollen abdomen, prolapse.
	22. Comb condition	Welfare challenges related to comb condition/damage/wounds/abnormalities/pecking wounds/temperature/colour/lesions/dehydration/dimensions, size/laterality.
(Ab)normal behaviour, activity, and welfare state	4. Behaviour and activity	Welfare challenges related to dust, sand and sunbathing, nesting, preening, grooming, play, daytime resting, (head) scratching, foraging, wing/leg stretching and flapping, body and tail shaking, bill wiping. Eating, drinking, litter/ground pecking, clustering, locomotion, stand, sit, moving, walking, jumping, litter exploring, daytime resting and sleeping, distance travelled, ambulating behaviour, foraging, head shaking, crouch, social interaction.
	7. Atypical egg laying	Welfare challenges related to floor eggs, double nest occupation, mislaid or aviary eggs, eggs outside nestboxes, out-of-nest eggs.
	12. Piling and smothering	Welfare challenges related to piling and smothering.
	13. Fearfulness, vigilance, flightiness and panic	Welfare challenges related to fear and panic, avoidance, escape, flightiness and fleeing, alert posture, freezing, vigilance behaviour. Excluding fear measurements performed using standardised behavioural tests (category 2).
	14. Nighttime sleeping and resting	Welfare challenges related to sleep during the dark hours. Includes any measurement performed during dark hours, overruling any other category as soon as the measurement was performed at night (e.g., vocalisation (category 21) at night).
	16. Perching behaviour and perch use	Welfare challenges related to daytime perch use, number of hens perching, resting and sleeping on perches during day, perch occupation.
	20. Flying, landing, falling, collisions, and kinematics	Welfare challenges related to flying, number of falls, 3D kinematics, accelerations during jumping and falling, behaviours associated with collisions, failed landings, distances of flights and jumps, peak force and contact area with perches, safe and unsafe landings.
	21. Vocalisations	Welfare challenges related to alarm/food/gakel/egg laying/fear/distress/panic/lonely calls, squawks of startle and pain, flock noise levels, latency to vocalise, stress audio signal and stress detection, warning vocalisations.
	24. Stereotypic behaviour/boredom	Welfare challenges related to pacing movements, circling movements, stereotyped behaviours.
Housing, environment, and monitoring	8. Location monitoring and environment use	Welfare challenges related to range use, enrichment use, nest use, free-range use, litter use, location during day/barn, nest box use, pop-hole use, ramp use, wintergarden use, veranda use, average travelled vertical distance, hen detection and count number of birds in cage, daytime resource occupancy, dust bath use, floor use, hen distribution, number of hens at feeders, use of aviary tiers, use of enrichment device, visits to substrate.
(Micro)predators and external threats	10. PRM infestation and comparable threats	Welfare challenges related to PRM infestation, presence of external parasites, ectoparasites, lice-mite infestation, mites on hen body and in traps.
	18. Predators	Welfare challenges related to cause of death, predator test, simulated hawk attack.
Residual	25. Other	Welfare challenges related to mortality, egg production, feed intake, mating and reproductive behaviours, pariah birds, pecking force, social network.

**Table 4:** Overview of quantitative questions included in the analysis of Topics 1 and 2 of the interactive TREP meeting.

Topic Number	Question Number	Question Type	Content
1	1	Ranking	What are the most prevalent unwanted behaviours?
1	2	Ranking	Which unwanted behaviours are easiest to recognise or detect by a human observer?
1	3	Ranking	What are the most threatening unwanted behaviours for hen longevity?
1	4	Ranking	Which unwanted behaviours are vital to detect as early as possible?
1	5	Ranking	Which unwanted behaviours take most time to deal with or cost the most money for laying hen farmers?
2	2	Multiple-choice	When it comes to [specific activity or task], do you believe protocols requiring one hour (or more) are more effective, or do you prefer using shorter checklists (less than [insert time])? Please share your reasoning in the discussion.
2	4	Multiple-choice	Is following up production parameters enough to generate an early warning for a particular behaviour? Please share your thoughts in the discussion.
2	5	Multiple-choice	Can we link production parameters and unwanted behaviours? Please share your reasoning in the discussion.
2	6	Multiple-choice	Is there a need for objective scoring using automated monitoring systems? Share your insights to enhance the discussion.
2	7	Multiple-choice	Is it practical to walk around the barn and observe/score animals while using a tablet or a smartphone? Please share your viewpoint to advance our discussion.

## Data Availability

The Slido-enabled PowerPoint presentation, the room-level outputs, the notetakers’ summaries for the TREP meeting and the double-blind data extraction overview are available in the Open Science Framework repository (https://osf.io/4hkx9/, accessed on 1 September 2026), together with the Excel file used to visualise Figure 2 and the PRISMA flow diagram.

## Abbreviations

The following abbreviations are used in this manuscript:

PRM	Poultry Red Mite
TREP	TransRegional Expert Panel
PLF	Precision Livestock Farming
OMELETTE	OptiMise and Extend hen Longevity to Expedite the Transition to susTainable Eggs
PTZ	Pan-tilt-zoom
PDCA	Plan–Do–Check–Act
TS	Topic search
RFID	Radio Frequency Identification
GPS	Global Positioning System
NWE	North-West Europe
SME	Small and Medium-sized Enterprise
NGO	Non-Governmental Organisation
Mbps	Megabits per second
CI/CD	Continuous Integration/Continuous Deployment
GPU	Graphics Processing Unit
YOLO	You Only Look Once
SOP	Standard Operating Procedure
